# Mutagenicity and carcinogenicity of combustion emissions are impacted more by combustor technology than by fuel composition: A brief review

**DOI:** 10.1002/em.22475

**Published:** 2022-03-25

**Authors:** David M. DeMarini, William P. Linak

**Affiliations:** ^1^ Air Methods and Characterization Division, Center for Environmental Measurement and Modeling U.S. Environmental Protection Agency Research Triangle Park North Carolina USA

**Keywords:** carcinogenicity, combustion emissions, complex mixtures, air pollution, emission factors, mutagenicity, open burning

## Abstract

Studies during the past 50 years have characterized the carcinogenicity and mutagenicity of extractable organic material (EOM) of particulate matter (PM) in ambient air and from combustion emissions. We have summarized conclusions from these studies and present data supporting those conclusions for 50 combustion emissions, including carcinogenic potencies on mouse skin (papillomas/mouse/mg EOM), mutagenic potencies (revertants/μg EOM) in the *Salmonella* (Ames) mutagenicity assay, and mutagenicity emission factors (revertants/kg fuel or revertants/MJ_thermal_) in *Salmonella*. Mutagenic potencies of EOM from PM in ambient air and combustion emissions span 1–2 orders of magnitude, respectively. In contrast, the revertants/m^3^ span >5 orders of magnitude due to variable PM concentrations in ambient air. Carcinogenic potencies of EOM from combustion emissions on mouse skin and EOM‐associated human lung cancer risk from those emissions both span ~3 orders of magnitude and are highly associated. The ubiquitous presence of polycyclic aromatic hydrocarbons (PAHs), nitroarenes, and aromatic amines results in mutagenic and carcinogenic potencies of PM that span only 1–3 orders of magnitude; most PM induces primarily G to T mutations. Mutagenicity emission factors of combustion emissions span 3–5 orders of magnitude and correlate with PAH emission factors (*r* > 0.9). Mutagenicity emission factors were largely a function of how material was burned (highly efficient modern combustors versus open burning) rather than what materials were burned. Combustion systems that minimize kinetic and mass‐transfer limitations and promote complete oxidation also minimize the mutagenicity of their emissions. This fundamental engineering principle can inform environmental and public health assessments of combustion emissions.

## 1 INTRODUCTION

Polluted air, water, and soil are the main environmental causes of deaths globally, and air pollution is among the primary environmental cause of deaths and disability globally (GBD 2016 Risk Factors Collaborators, 2017). The primary contributor to air pollution is combustion emissions from a wide variety of sources (Russell, 2013). Combustion is a complex progression of physical and chemical processes involving the mass transfer and exothermic chemical reactions of a fuel (reductant, usually carbonaceous, such as natural gas, oil, coal, wood, gasoline, etc.) and an oxidant (usually atmospheric oxygen in excess). These interactions result in a highly reactive flow with fast heat and mass transfer. High temperatures sustain hundreds to thousands of elementary reactions involving stable species and free radicals. Free radical chemistry is critical for the initiation, propagation, and termination of flame reactions.

Thermodynamically, for hydrocarbon fuels, chemical equilibrium overwhelmingly favors the formation of carbon dioxide (CO_2_) and water (H_2_O) as reaction products. Unfortunately, complete combustion (full oxidation) is almost impossible to achieve due to kinetic and mass transfer limitations. Incomplete oxidation leads to the formation of carbon monoxide (CO), a large variety of volatile, semi‐volatile, and non‐volatile organic species, as well as carbon particulate (soot). To minimize the formation of these products of incomplete combustion, equipment is often designed in attempts to maximize the time, temperature, and turbulence of reactants within flame and high‐temperature post‐flame environments in order to minimize kinetic and mass transfer limitations. These products of incomplete combustion are the main component of combustion emissions that comprise most of the mutagenic and carcinogenic organics in polluted air and form the basis for this review.

The emissions from three general classes of technologies have been evaluated for mutagenicity and carcinogenicity: (1) highly efficient technologies, such as utility power plants, which involve high temperatures, extensive controlled mixing of fuel and oxygen, and adequate oxygen; (2) technologies with intermediate efficiency, such as internal combustion engines; and (3) low‐level technology open burning, exemplified by the majority of biomass cookstoves, wildfires, structural fires, and burn pits, which involve low temperatures and poor mixing of fuel and oxygen resulting in inadequate amounts of oxygen (where needed) to sustain combustion. All can produce a wide array of mutagenic and carcinogenic emissions that can contribute to air pollution, but their emissions and subsequent mutagenic and carcinogenic effects can vary by orders of magnitude.

Air pollution is associated with an array of environmental (IPCC, 2021) and health effects, including prenatal and children's health (Heft‐Neal et al., 2022; Johnson et al., 2021), neurological effects (Kim et al., 2020; Shaffer et al., 2021), cardiovascular disease (Lewtas, 2007), and cancer (IARC, 2016). Although people are exposed to both the particulate and gas phase of air pollution and combustion emissions, most epidemiology and experimental carcinogenesis and mutagenesis studies have linked these health effects to the particulate matter (PM), especially PM that is ≤2.5 μm in diameter (PM_2.5_). Carbonaceous components of PM are products of incomplete combustion, and as described below, the organics bound to PM are the portion of PM most associated with the mutagenicity and carcinogenicity of combustion emissions and air pollution.

The mutagenicity and carcinogenicity of the extractable organic material (EOM) from PM, especially PM_2.5_, have been studied extensively for decades, as chronicled in many reviews (Claxton, 2014a, 2014b; Claxton, 2015a, 2015b, 2015c; Claxton et al., 2004; Claxton & Woodall Jr., 2007; IARC, 1986, 1989, 2004, 2010c, 2012, 2014, 2016; Lewtas, 1988). These studies have identified some of the main chemical classes in the PM and the mechanisms by which PM causes a range of health effects. In addition, these studies have highlighted the sources of combustion emissions and the various ways by which an array of materials are burned, leading to potential health effects.

Several general conclusions have emerged from these studies, and we have consolidated them in this review. To illustrate these conclusions, we present data on the extractable organics from PM of 50 combustion emissions, including the association between carcinogenic potencies in humans as determined by epidemiology and experimental carcinogenic potencies in rodents, mutagenic potencies in *Salmonella*, and mutagenicity emission factors in *Salmonella* for PM from ambient air and a wide array of combustion emissions. These data demonstrate several fundamental features of PM‐associated mutagenicity and carcinogenicity that can inform assessments of environmental and health effects of air pollution and combustion emissions.

## 2 CARCINOGENIC POTENCIES OF ORGANICS FROM COMBUSTION EMISSIONS AND ASSOCIATION WITH HUMAN LUNG CANCER RISK

The first report of the experimental carcinogenicity (in mice) of organics from polluted air was by Leiter et al. (1942). Since then, numerous studies have confirmed this observation in experimental animals, as reviewed by Claxton and Woodall (2007) and IARC (2016). Based on a range of data, outdoor air pollution and PM in outdoor air pollution are Group 1 (known) human lung carcinogens (IARC, 2016). A variety of combustion emissions that contribute to varying extents to the carcinogenicity of polluted air have also been evaluated as known human carcinogens (IARC, 2012), including diesel exhaust (IARC, 1989, 2014), cigarette smoke (IARC, 1986; IARC, 2004), and household combustion of coal (IARC, 2010c). Emissions from the burning of biomass (primarily wood) have been evaluated as a Group 2A (probable) human carcinogen (IARC, 2010c).

**FIGURE 1:**
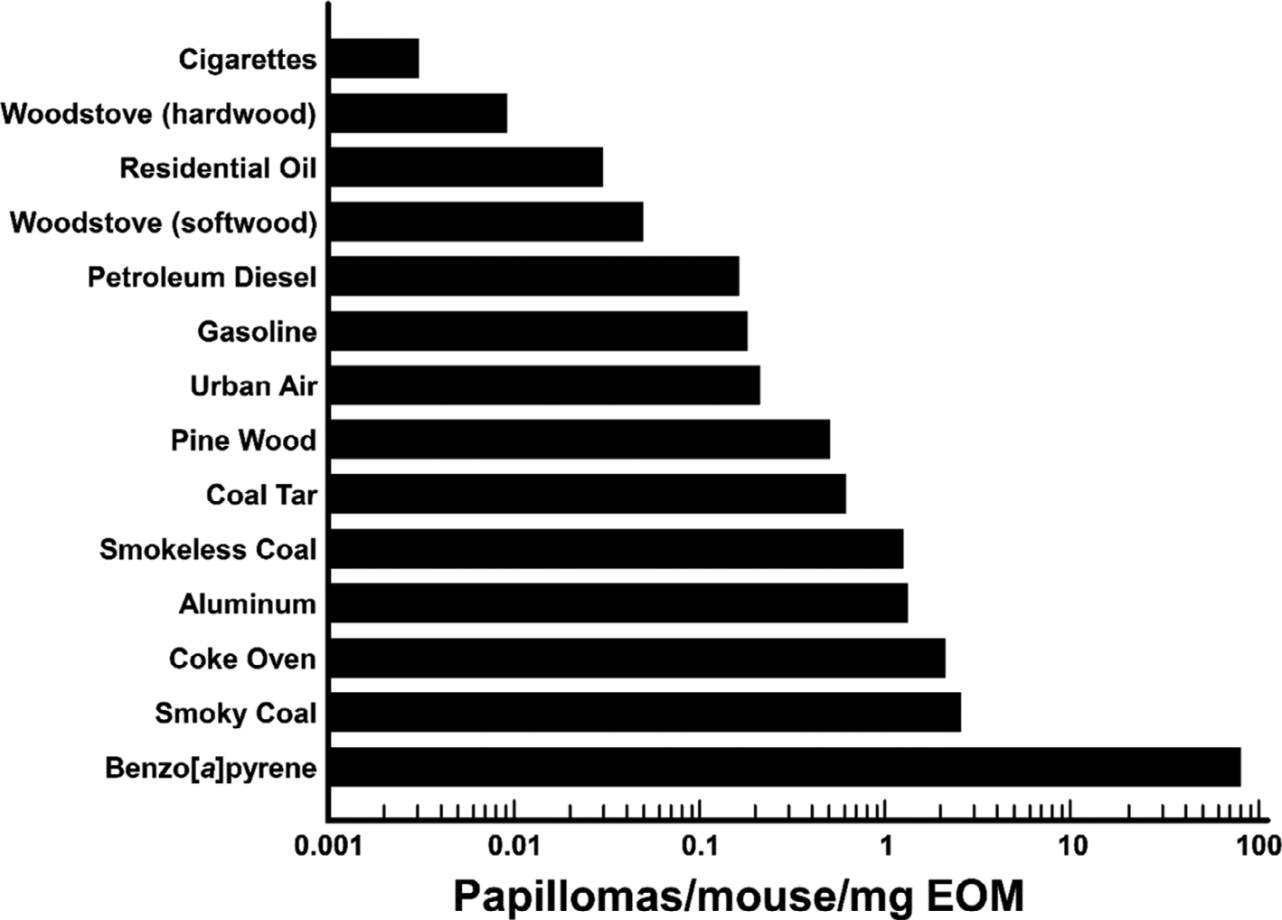
Carcinogenic potency of extractible organic material (EOM) on Sencar mouse skin from a variety of combustion emissions, coal tar, and benzo[*a*]pyrene. Data for smokeless coal, smoky coal, pine, and benzo[*a*]pyrene are from Mumford et al. (1990); the remaining data are from DeMarini and Lewtas (1995). Error measurements were not provided in the cited papers except in Mumford et al. (1990), preventing an overall analysis of significant differences among the various complex mixtures or B[*a*]P

Figure 1 summarizes the carcinogenic potency on mouse skin of the EOM (primarily from PM_2.5_) from a variety of combustion emissions and urban air. In this assay, the organics were painted onto the skin of mice, and after typically 6 months, the number of lesions (papillomas) on the skin were counted; thus, the carcinogenic potency was expressed as the number of papillomas/mouse/mg EOM. In nearly all of these studies, histopathological analyses showed that the papillomas were carcinomas, that is, malignant tumors, rather than benign tumors.

Coal tar is technically not a combustion emission but a liquid pyrolysis byproduct, produced by thermally induced devolatilization and partial distillation of coal in the absence of oxygen to produce solid coke. However, we have included it because it is among the complex mixtures for which there are carcinogenicity data in this assay. The carcinogenic potencies of these organic extracts span ~3 orders of magnitude, with one of the most important human lung carcinogens, cigarette smoke, being the weakest among these extracts. The Group 1 human carcinogen benzo[*a*]pyrene (B[*a*]P) (IARC, 2010a) was included as a positive control in most of these studies because polycyclic aromatic hydrocarbons (PAHs) such as B[*a*]P were shown to be a primary class of carcinogen in these extracts. The carcinogenic potency of this single compound extends the potency range an additional order of magnitude beyond that of the organic extracts.

In contrast to the ~3 orders of magnitude range of carcinogenic potencies seen for the complex mixtures associated with combustion‐related emissions, the carcinogenic potencies of 1298 compounds in mice and rats span >10‐million‐fold (>7 orders of magnitude) (Gold & Zeiger, 1997). The basis for this difference is explored later in the section on PAHs; however, likely explanations are that the mutagenicity of combustion emissions and PM is largely due to the presence of just a few chemical classes, especially PAHs, aromatic amines, and nitro‐PAHs. Thus, having a rather similar mix of compounds likely results in a rather similar range of mutagenic potencies.

Although cigarette smoke, outdoor air pollution, diesel exhaust, and the emissions from the indoor combustion of coal, particularly smoky coal (a low‐quality fossil fuel with high volatile organic content) (Mumford et al., 1990), are Group 1 human carcinogens (IARC, 2004, 2010c, 2014, 2016), the carcinogenic potencies of the organic extracts from their PM on mouse skin are not the same. Thus, the carcinogenic potency of the complex mixture of organics from smoky coal emissions is 1000 times greater than that from cigarette smoke, with the potencies of diesel exhaust and urban air in between (Figure 1). Chemical analyses have shown that these various organic extracts consist of thousands of compounds; however, bioassay‐directed fractionation and other methods have indicated that much of the carcinogenicity and mutagenicity of these organic extracts are largely due to a few primary chemical classes, such as PAHs, aromatic amines, and nitroarenes (nitro‐PAHs) (Hecht & DeMarini, 2019; IARC, 1986, 2004, 2010b, 2010c; IARC, 2012, 2014, 2016).

**FIGURE 2:**
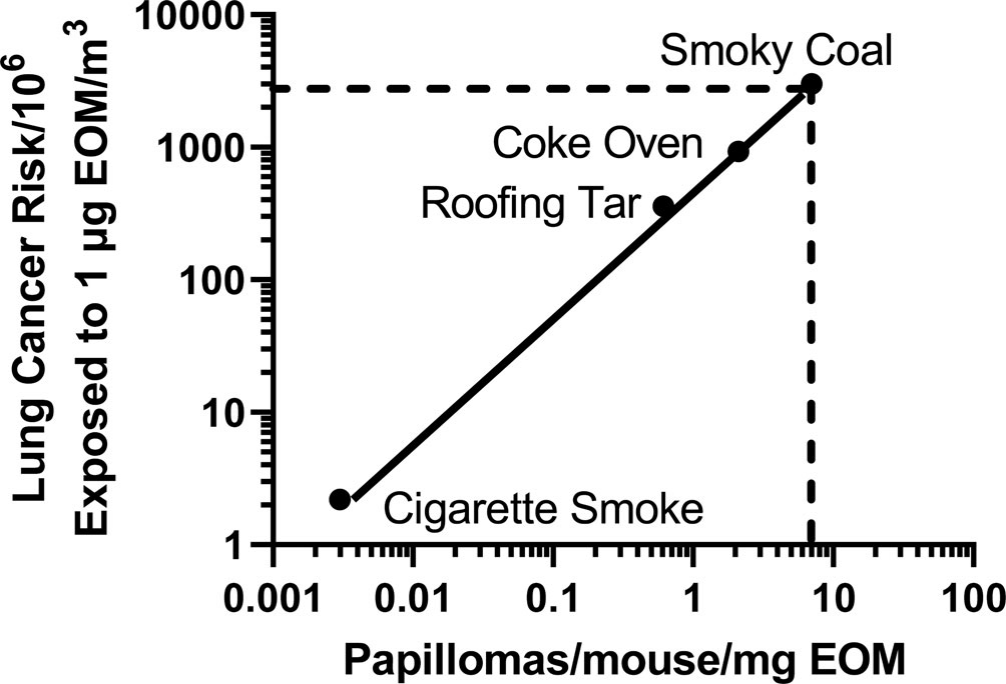
Association between human lung cancer risk and carcinogenic potency on mouse skin of the EOM from PM of various combustion emissions. Human lung cancer data are from Albert et al. (1983); mouse tumor potencies for cigarette smoke, roofing tar, and coke oven are from Lewtas (1993). Mouse skin carcinogenic potency for smoky coal is from Mumford et al. (1990) and was extrapolated (dashed line) to estimate human lung cancer risk. Figure modified and redrawn from Cupitt et al. (1994) to include smoky coal, and the solid line is a linear regression. Error measurements were not provided in the cited papers except in Mumford et al. (1990), preventing an overall analysis of significant differences among the samples

Figure 2 illustrates the association between the carcinogenic potencies of some of these organic extracts on mouse skin and the unit lung cancer risk in humans exposed to such organics.

Both the rodent carcinogenic potency for these extracts and the human lung cancer risk span 3 orders of magnitude. The strong association between the two endpoints suggests that these complex organic extracts exhibit similar tumor potencies in both species. As noted by Cupitt et al. (1994), such an association permits the extrapolation of the mouse skin carcinogenic potency to human lung cancer risk for samples where epidemiologic studies are not possible but where air or combustion emission samples could be collected and tested for their carcinogenic potency on mouse skin.

We show an example of this with smoky coal, whose EOM has been evaluated for its carcinogenic potency on mouse skin, but for which lung cancer relative to EOM exposure has not been determined (Figure 2). Women exposed to indoor smoky coal emissions have some of the highest frequencies of lung cancer among never smokers in the world (Mumford et al., 1987). Using the comparative‐potency data in Figure 2, an extrapolation based on the mouse papilloma data suggests that the EOM from smoky coal may induce ~3000 lung cancer cases per million people exposed at 1 μg EOM/m^3^ of inhaled air for 70 years.

## 3 MUTAGENIC POTENCIES OF ORGANICS FROM AIR AND COMBUSTION EMISSIONS

In this review, mutagenic potency refers to the number of mutant colonies (revertants or rev) produced in the *Salmonella* (Ames) mutagenicity assay per either μg of EOM, μg of PM, or m^3^ of air. The mutagenicity of the EOM from the PM of ambient air was first reported in 1977 (Pitts et al., 1977; Talcott & Wei, 1977; Tokiwa, 1977) and has since been followed by hundreds of additional studies (Claxton et al., 2004; Claxton & Woodall Jr., 2007; IARC, 2016). The 1970s and 1980s also marked the first reports of the mutagenicity of a variety of combustion emissions, including diesel exhaust (Claxton, 2015a; IARC, 1989, 2014); cigarette smoke (IARC, 1986, 2004); and combustion of coal, wood, and other types of biomass (IARC, 2010c). Reviews of hundreds of studies have shown that people with elevated levels of genotoxicity biomarkers (chromosome aberrations, micronuclei, DNA damage measured by ^32^P‐postlabeling or the comet assay, DNA methylation, telomere shortening, etc.) associated with exposure to ambient air pollution, especially traffic‐impacted air, are at increased risk for cancer (DeMarini, 2013; Demetriou et al., 2012). These studies highlight the role of mutagenic combustion emissions to air pollution, resulting in the induction of a variety of genotoxicity biomarkers.

**FIGURE 3:**
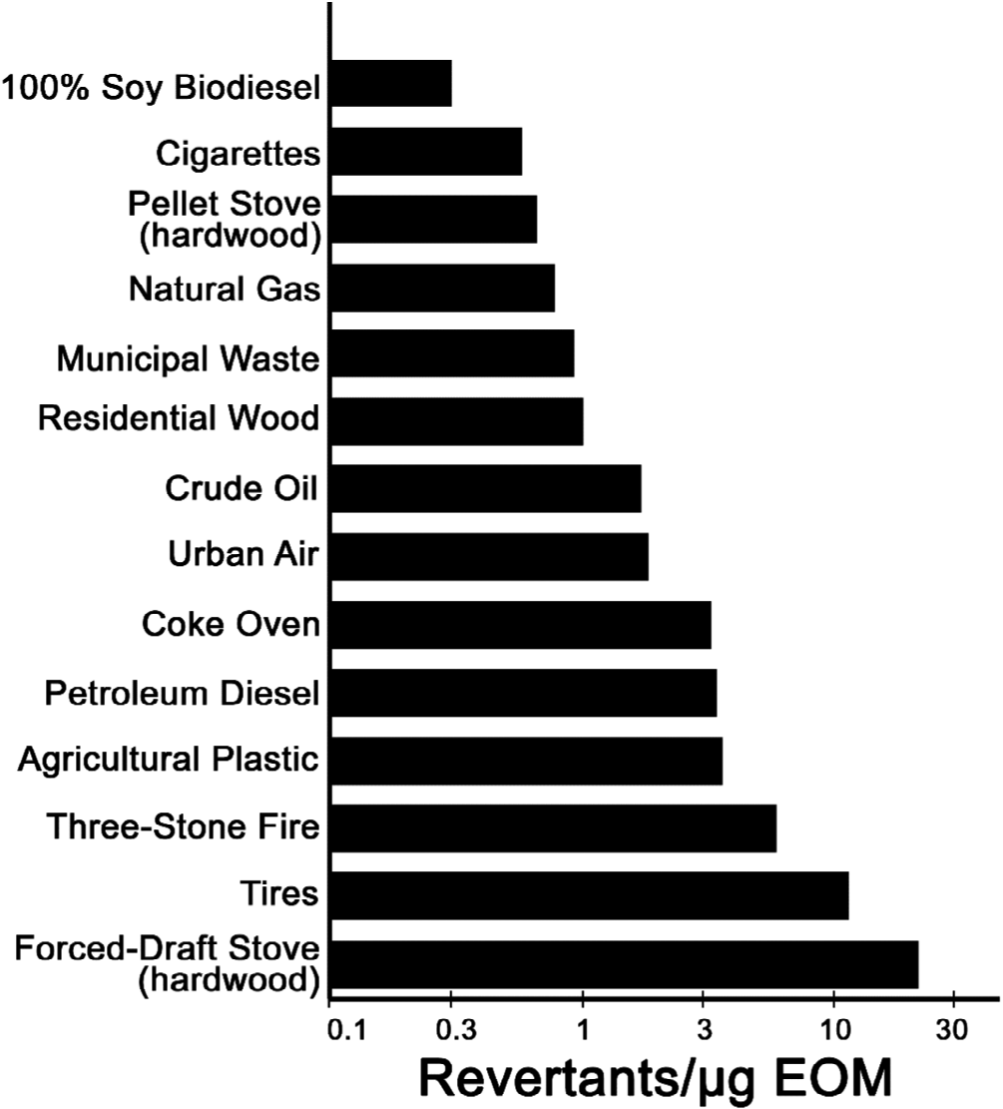
Mutagenic potency of EOM in *Salmonella* TA98 + S9 from a variety of combustion emissions; data are from Table 1, except for cigarettes, which are 2R4F reference cigarettes (DeMarini et al., 2008) and urban air (Maselli et al., 2019). The three‐stone fire and forced‐draft stove are described in Mutlu et al. (2016). Error measurements were available for 100% soy biodiesel, crude oil, three‐stone fire, and forced‐draft fire, but not for the other complex mixtures, preventing an overall analysis of significant differences among the samples

**FIGURE 4:**
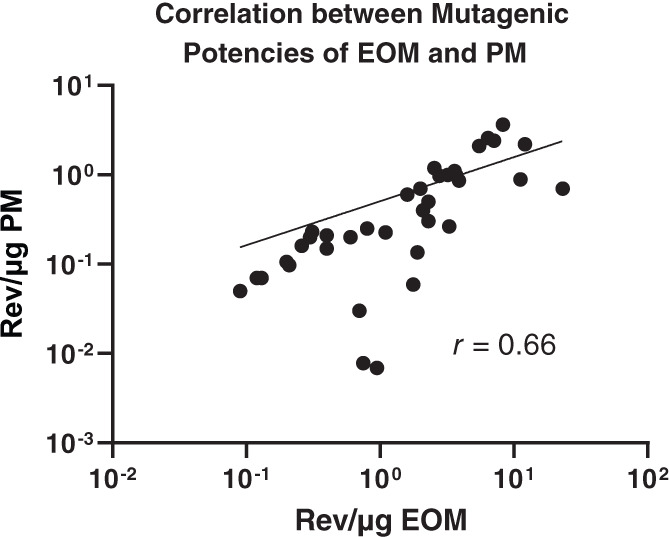
Correlation between the mutagenic potencies of EOM and PM. Data from Table 1; however, those for toluene and polyethylene from the rotary kiln study were not included as explained in the text

Table 1 shows the %EOM, mutagenic potencies of EOM and PM, and the PM and mutagenicity emission factors for 50 combustion emissions; Figure 3 shows a histogram of a subset of these data. Using the data from Table 1 except those for the two rotary kiln studies as explained below, the *x*‐axis of Figure 4 shows that the mutagenic potencies per μg of EOM span 2 orders of magnitude (~0.2 to ~20 rev/μg EOM). Despite the fact that these emissions came from varied sources and were generated by different technologies, that is, by the highly efficient and controlled combustion within utility boilers versus minimally efficient combustion as exemplified by open burning, such as agricultural plastic, three‐stone fire (three stones on the ground supporting a pot under which there is a fire), tires, and so forth, the mutagenic potencies of their EOMs span a rather narrow range compared to the range of mutagenic potencies of a group of 300 single chemicals, which span >6 orders of magnitude (McCann & Ames, 1975).

**TABLE 1:** Mutagenic potencies and mutagenicity emission factors for various combustion emissions^a^

Source	%EOM	Rev/μg EOM	Rev/μg PM	mg PM/m^3^	Rev/m^3^	mg PM/kg fuel	Rev/kg fuel	Rev/MJ_th_	References
Utility power plants									
Oil							3,000	70	Lewtas (1988)
Coal		0.5–9.4					6,000	230	Lewtas (1988), DeMarini and Lewtas (1995)
Wood							20,000	1,000	Lewtas (1988)
Residential heating									
Oil		2–5					1,000,000	2,500	Lewtas (1988), DeMarini and Lewtas (1995)
Wood		1					5,000,000	250,000	Lewtas (1988), DeMarini and Lewtas (1995)
Incineration									
Municipal waste	0.73	0.95	0.0069	43.8	304	63,400	440,000	38,000	Watts et al. (1992)
Medical/path waste	1.0	0.75	0.0078	59.6	465	8,970	70,000	580	Watts et al. (1992)
Pesticide (dinoseb)									
Staged	3.3	1.77	0.059	130.3	7,700	1,610	95,000	3,100	DeMarini et al. (1991), Linak et al. (1991)
Staged + reburn	8.1	3.28	0.26	71.9	19,000	862	228,000	6,600	DeMarini et al. (1991), Linak et al. (1991)
No. 2 Fuel oil									
Staged	38	0.40	0.15	3.2	483	79	12,000	270	DeMarini et al. (1991), Linak et al. (1991)
Staged + reburn	47	0.21	0.10	4	391	71	7,000	140	DeMarini et al. (1991), Linak et al. (1991)
Rotary kiln^b^									
Natural gas	31	0.8	0.25			4	1,000	20	DeMarini et al. (1992)
Toluene	0.26	20.1	0.05			12,300	640,000	14,000	DeMarini et al. (1992)
Polyethylene	0.03	400	0.12			7,480	900,000	19,000	DeMarini et al. (1992)
Vehicle engines									
Gasoline/catalyst^c^	8.6						100,000	2,200	Lewtas (1988), DeMarini and Lewtas (1995)
Gasoline/non‐catalyst^c^							1,000,000	18,000	Lewtas (1988)
Diesel trucks/buses^d^		1.4–15.1					4,000,000	88,000	Lewtas (1988), DeMarini and Lewtas (1995)
Diesel vehicles^d^		1.4–15.1					4,000,000	88,000	Lewtas (1988), DeMarini and Lewtas (1995)
150‐kW Diesel bus^e^							140,000	3,000	Mutlu et al. (2015b)
4.3‐kW Diesel generator									
100% Diesel fuel	26	3.7	1	27.96	28,000	1,910	1,910,000	40,000	Mutlu et al. (2015a), Mutlu et al. (2015b)
Diesel +20% soy	21	2.1	0.4	24.00	9,600	1,660	670,000	20,000	Mutlu et al. (2015a), Mutlu et al. (2015b)
Diesel +50% soy	32	0.6	0.2	24.85	4,970	1,640	330,000	10,000	Mutlu et al. (2015a), Mutlu et al. (2015b)
100% Soy biodiesel	60	0.3	0.2	19.71	3,940	1,220	240,000	10,000	Mutlu et al. (2015a), Mutlu et al. (2015b)
Diesel +5% canola	30	3.2	1	15.9	15,900	426	430,000	9,000	DeMarini et al. (2019)
Diesel +20% canola	23	2.3	0.5	20	10,000	544	270,000	6,000	DeMarini et al. (2019)
Diesel +50% canola	31	3.6	1.1	18	19,800	466	510,000	12,000	DeMarini et al. (2019)
100% Canola biodiesel	35	2.0	0.7	15	10,500	355	250,000	6,000	DeMarini et al. (2019)
100% Waste veg oil	40	1.6	0.6	13.1	7,860	310	190,000	5,000	DeMarini et al. (2019)
Cookstoves									
Three‐stone fire	34	7.1	2.4			1,540	3,680,000	240,000	Mutlu et al. (2016)
Natural‐draft stove	18	12.1	2.2			920	2,000,000	120,000	Mutlu et al. (2016)
Forced‐draft stove	3	23.2	0.7			454	320,000	20,000	Mutlu et al. (2016)
Pellet‐fueled gasifier									
Hardwood	5.3	0.7	0.03			339	10,000	500	Champion et al. (2020)
Peanut hulls	24	1.1	0.23			79	20,000	1,500	Champion et al. (2020)
Open burning									
Crude oil	7	1.9	0.14			58,000	7,810,000	190,000	DeMarini et al. (2017), Gullett et al. (2017)
Tires^f^	7.9	11.2	0.88	1.12	993	108,000	95,500,000	2,740,000	DeMarini et al. (1994a), Lemieux et al. (1998)
Simulated open burning									
Kerosene	13	2.3	0.30	3.55	644	51,300	15,600,000	416,000	Linak et al. (1989)
Agricultural plastic^g^									
Clean pile	22	3.9	0.86	16.1	6,950	26,900	23,200,000	521,000	Linak et al. (1989)
Clean forced air	53	0.4	0.21	9	2,810	45,300	9,500,000	213,000	Linak et al. (1989)
Used pile	53	0.2	0.11	4.43	1,170	42,300	4,500,000	101,000	Linak et al. (1989)
Used forced air	73	0	0	4.16	0	0	0	0	Linak et al. (1989)
Red oak									
Smoldering	50	0.1	0.07	973	126,000	65,500	8,520,000	392,000	Kim et al. (2018)
Flaming	47	2.5	1.19	8	20,300	468	1,190,000	55,000	Kim et al. (2018)
Peat									
Smoldering	73	0.3	0.23	488	151,000	51,800	16,100,000	699,000	Kim et al. (2018)
Flaming	38	6.0	2.09	15	82,500	380	2,090,000	91,000	Kim et al. (2018)
Pine needles									
Smoldering	62	0.3	0.16	624	162,000	60,800	15,800,000	1,321,000	Kim et al. (2018)
Flaming	35	2.8	0.97	18	50,000	349	970,000	81,000	Kim et al. (2018)
Pine									
Smoldering	60	0.1	0.07	1,050	126,000	85,800	10,300,000	515,000	Kim et al. (2018)
Flaming	43	8.3	3.65	1	8,290	440	3,650,000	182,000	Kim et al. (2018)
Eucalyptus									
Smoldering	52	0.1	0.05	1,420	128,000	83,200	7,490,000	389,000	Kim et al. (2018)
Flaming	40	6.4	2.58	10	64,400	401	2,580,000	134,000	Kim et al. (2018)

^a^ Values are from the cited papers, or we calculated the values from data in the papers; values are expressed at 2–3 significant figures.

^b^ Rotary kiln studies were pilot‐scale tests that examined transient emissions where all the oxygen was depleted during a short time.

^c^ We calculated the rev/MJ_th_ from the published rev/kg fuel value using the assumed typical higher heating value for gasoline (45.4 MJ/kg) obtained from GREET (2010).

^d^ We calculated the rev/MJ_th_ from the published rev/kg fuel value using the assumed typical higher heating value for diesel (45.6 MJ/kg) obtained from GREET (2010).

^e^ We calculated the rev/kg fuel from the published rev/MJ_th_ using the fuel value using the assumed typical higher heating value for diesel (45.6 MJ/kg) obtained from GREET (2010).

^f^ Average of two experiments of chunked tires, i.e., tires cut into 1/4th or 1/6th.

^g^ The plastic is polyethylene plastic sheets; values are the sum of the data from the prefilter and postfilter; used plastic was exposed to pesticides and soil.

Figure 4 does not contain Table 1 data from two small pilot‐scale rotary kiln studies examining transient introduction of polyethylene and contained toluene charges because, due to the extremely high temperatures and temporary absence of available oxygen, we believe that these studies resulted primarily in PM more similar to carbon black rather than to normal combustion emissions. Table 1 indicates that the %EOM for these PM samples are low, although what little EOM is present is highly mutagenic. Carbon black is an industrial product typically produced through the sub‐stoichiometric combustion of oil‐based fuels. High reactor temperatures optimize the formation of crystalline elemental carbon agglomerates with low condensed organic carbon. Carbon black has low %EOM and mutagenicity, and when mutagenicity was detected, it was associated with extractable nitropyrene contaminants (Rosenkranz et al., 1980), likely due to high temperature organic reactions with fuel‐bound or atmospheric nitrogen.

Using the data in Table 1, Figure 4 shows a moderate correlation (*r* = .66) between the mutagenic potencies of the EOM and the PM for a variety of combustion emissions, with both sets of values varying by ~2 orders of magnitude. Thus, despite the well‐documented variation in chemical composition of PM from ambient air from different airsheds and from different combustion sources, the mutagenic potencies of these complex mixture PM samples span a rather narrow range of mutagenic potencies compared to that of a set of single compounds.

As bioassay‐directed fractionation studies have shown, this is most likely because just a few chemical classes (e.g., PAHs, nitroarenes, and aromatic amines) can account for much of the mutagenic activity of a diversity of PM samples, such as those from diesel exhaust (IARC, 2014; Mutlu et al., 2013, 2015a), cigarette smoke (IARC, 1986, 2004), incinerator emissions (DeMarini et al., 1996), tire fire emissions (Lemieux et al., 1998), and urban air (IARC, 2016). The ubiquitous presence of some of these classes of mutagens may account for the relatively narrow range of mutagenic potencies of PM among combustion emissions.

Consistent with these results from combustion emissions are data for PM from ambient air pollution. Based on an analysis of data from >250 published studies involving >3000 air samples from five continents, the mutagenic potencies of the EOM from ambient air PM span only ~1 order of magnitude, whereas the mutagenicity of air per cubic meter spans >5 orders of magnitude (IARC, 2016). Thus, the wide range of air mutagenicity worldwide is due not so much to the intrinsic variation in the mutagenic potency of the PM itself but, rather, to the amount (concentration) of PM per cubic meter of air, which varies considerably worldwide. A recent study designed to illustrate this concept using identical methods for extracting and testing organics from air PM from three cities confirmed that the mutagenic potencies of the EOM from the PM_2.5_ of air from cities on three different continents (Kyoto, Japan; Stockholm, Sweden; and Limeira, Brazil) varied only ~2‐fold, whereas the mutagenicity of the air expressed per cubic meter varied ~20‐fold (Maselli et al., 2019).

The relatively narrow range of mutagenic potencies of EOM and PM from air or combustion emissions (1–2 orders of magnitude) contrasts with the somewhat wider range (3 orders of magnitude) of carcinogenic potencies for many of the same EOM samples (Figure 1). This is likely due to several reasons. One is because carcinogenesis is a multi‐step process, with mutagenesis being only one of those steps. Chemical components other than mutagens in these organic extracts, such as those that might alter gene expression, also play a role in the carcinogenicity of these extracts (IARC, 2004, 2010c, 2014, 2016), accounting for a broader range of carcinogenic potencies relative to mutagenic potencies for these organic extracts. A second reason is the nature of the two assays; one is a bacterium, and the other is a rodent, with the two having vastly different biology, including different adsorption, distribution, metabolism, and excretion (ADME).

## 4 PM‐ASSOCIATED MUTAGENICITY EMISSION FACTORS OF COMBUSTION EMISSIONS

**FIGURE 5:**
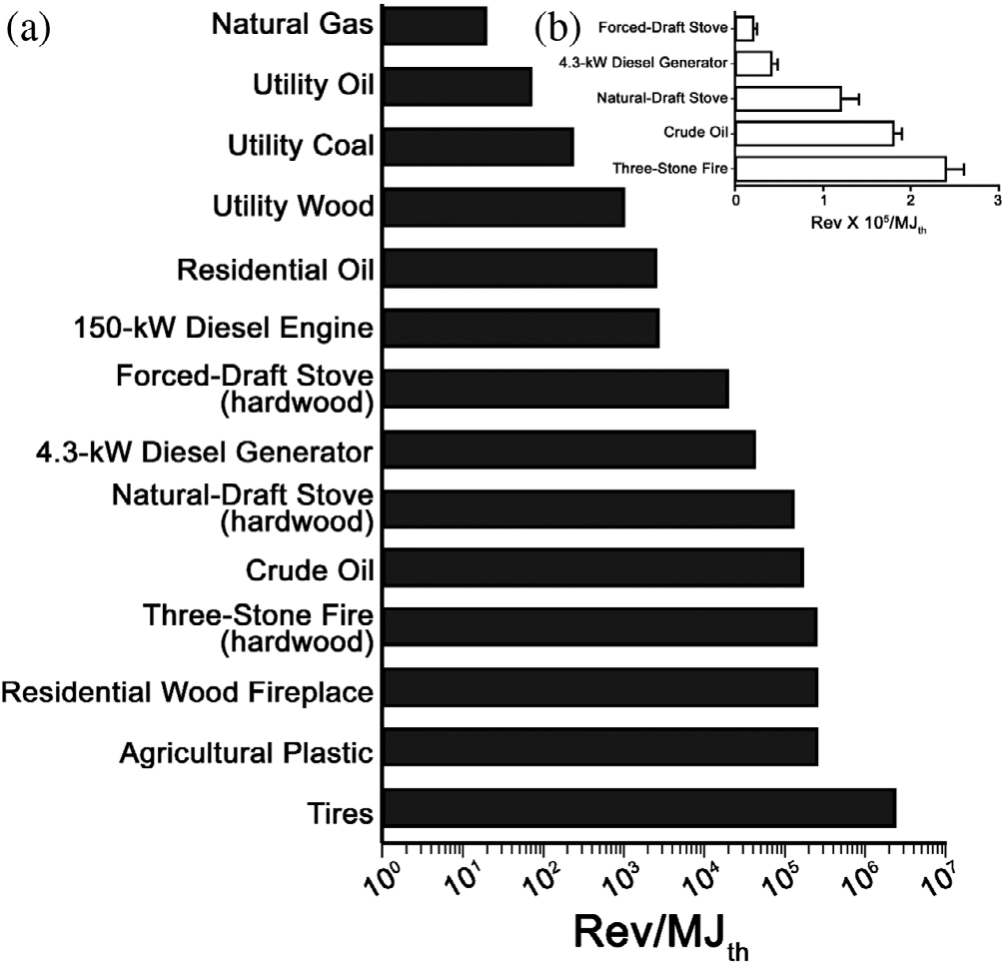
Mutagenicity emission factors in TA98 + S9 for a variety of emissions. (a) Data plotted on a log scale; (b) subset of the data replotted on a linear scale showing standard error values; these mutagenicity emission factors were significantly different from one another (*p* < .05). The figure is modified from DeMarini et al. (2017). In general, the relative values are similar regardless of strain of *Salmonella*, as evidenced for four different diesel emissions in 16 strain/S9 combinations (Mutlu et al., 2015c). Other than for the samples noted in (b), error values were not generally available for the other samples, preventing an overall analysis of significant differences among the samples

An emission factor is a measure of how much pollution is produced and emitted per some unit of activity. Although only a few papers have reported the mutagenicity emission factors for combustion emissions, such a metric allows comparison among diverse sources. Table 1 lists the mutagenicity emission factors for a variety of combustion emissions expressed in terms of the number of *Salmonella* revertants (rev) per either the mass of fuel burned (kg fuel) or, based on the calorific heating value of the fuel, the thermal energy released (megajoule_thermal_ [MJ_th_]); Figure 5 illustrates a sub‐set of these data. These two ways of expressing the mutagenicity emission factor are interchangeable (related by the measured heating values for the various fuels) and, using the data in Table 1, highly correlated (*r* = .96). The details regarding the scales and technologies associated with these combustion studies were not always described in the papers; however, the samples included large industrial and utility boilers (presumably with PM controls), residential heating, various types of incineration, a small pilot‐scale rotary kiln incinerator, gasoline and diesel internal combustion engines, different types of cookstoves, and the open burning or simulation of open burning of a variety of materials.

Natural gas had the lowest mutagenicity emission factor, and that was from a study featuring steady‐state combustion and high levels of excess air to maintain high temperatures within a small pilot‐scale rotary kiln incinerator (DeMarini et al., 1992). In addition, oil, coal, or wood burned in large (presumably relatively efficient) industrial and utility boilers also had relatively low mutagenicity emission factors. Internal combustion engines (gasoline and diesel) and engineered cookstoves had intermediate values, and the highest values were for low‐technology wood combustion (three‐stone fire and residential fireplace) and open burning, with tire fires having values exceeding 10^6^ rev/MJ_th_.

**FIGURE 6:**
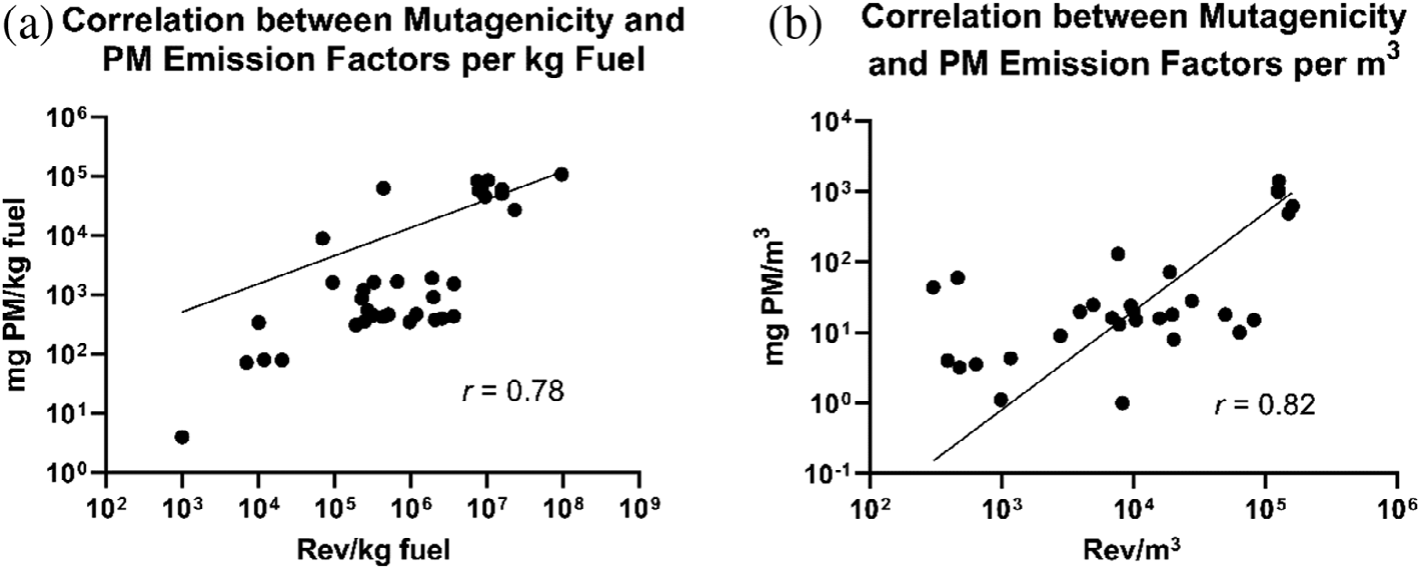
Correlation between mutagenicity and PM emission factors expressed per (a) kg fuel and (b) PM concentrations expressed per m^3^. Data from Table 1; however, those for toluene and polyethylene from the rotary kiln study were not included as explained in the text

Based on data from a wide variety of combustion emissions (Table 1), the mutagenicity and PM emission factors were correlated at *r* = .78 when expressed per kg fuel (Figure 6a), and the mutagenicity and PM concentrations were correlated at *r* = 0.82 when expressed per cubic meter (Figure 6b). Collectively, these correlations show that the mutagenicity emission factors for a variety of combustion emissions span ~3 orders of magnitude and are largely influenced by the amount of PM rather than the source or mutagenic potency of the PM. This is similar to findings for ambient air, whose mutagenicity spans >5 orders of magnitude in terms of rev/m^3^ and where the mutagenicity of the air per cubic meter is determined largely by the amount of PM in the air rather than the intrinsic mutagenic potency of the PM itself (IARC, 2016). The amount of PM emitted is largely a function of how the material is burned, not what is burned. Consequently, the technology used to burn material typically has more of an impact on the mutagenicity emission factor than does the type of material being burned.

**FIGURE 7:**
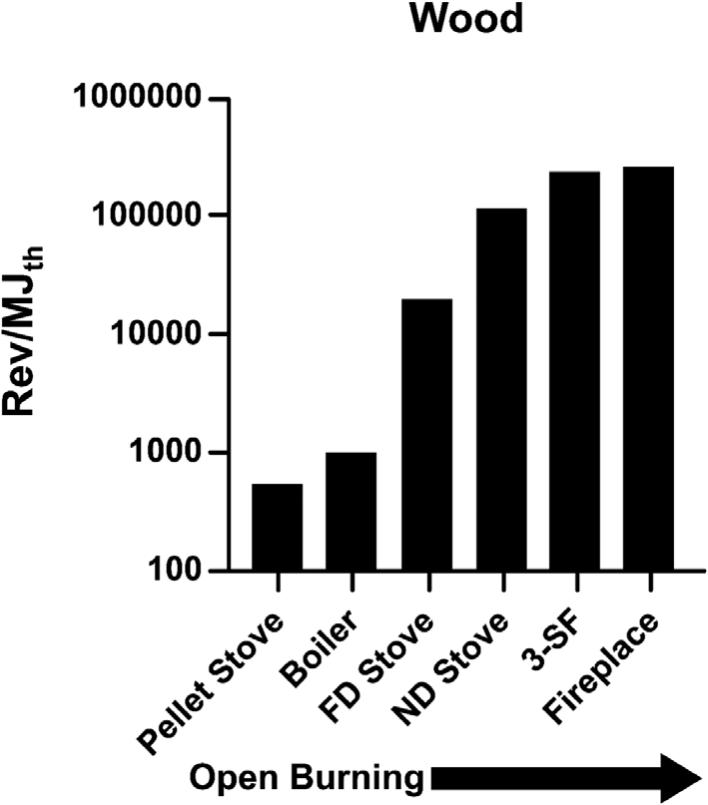
Mutagenicity emission factors in *Salmonella* TA98 + S9 for wood burned in a variety of ways, going from controlled combustion on the left to more open burning on the right. Data for pellet stove from Champion et al. (2020); for boiler and fireplace from DeMarini and Lewtas (1995); and for forced‐draft (FD) stove, natural‐draft (ND) stove, and three‐stone fire (3‐SF) from Mutlu et al. (2016). Error measurements were not reported for the boiler and fireplace, preventing an overall analysis of significant differences among the samples

This conclusion is illustrated for wood in Figure 7, which shows the rev/MJ_th_ for wood burned six different ways, with a pellet‐fueled gasifier stove and an industrial or utility boiler having the lowest values, a forced‐draft stove (equipped with an internal fan to promote improved fuel/air mixing) having an intermediate value, and the rest (natural draft stove, three‐stone fire, and conventional home fireplace) largely using natural buoyancy forces for fuel/air mixing, having the highest values. Thus, depending on the combustion technology used, the combustion of similar wood (oak in nearly all of these studies) can produce mutagenicity emission factors that span 3 orders of magnitude.

Viewed the other way, a study in which identical combustion conditions were used to burn a variety of types of wood (pine, oak, and eucalyptus) as well as forest duff (peat and pine needles), reflective of the predominant type of biomass in different wildland fires, found that the resulting mutagenicity emission factors under smoldering conditions were not significantly different (Kim et al., 2018). Wildfires (as well as structural fires) are classic examples of open burning, and although the mutagenicity emission factors may be similar among different types of biomass (Kim et al., 2018), exposure levels may vary, resulting in different levels of genotoxicity in wildland firefighters (Adetona et al., 2019; Wu et al., 2021).

As shown in Table 1 and Figure 5, open burning (uncontrolled combustion) generally results in a similar range of mutagenicity emission factors (10^5^–10^6^ rev/MJ_th_) regardless of what is being burned. This was confirmed by a recent study in which open burning was simulated for a variety of materials (wood, plastic, cardboard, etc.) used as fuel in burn pits found on U.S. military bases in Iraq and Afghanistan (Kim et al., 2021). The authors found that under similar smoldering conditions, there were no significant differences among the mutagenicity emission factors for these different types of fuels.

**FIGURE 8:**
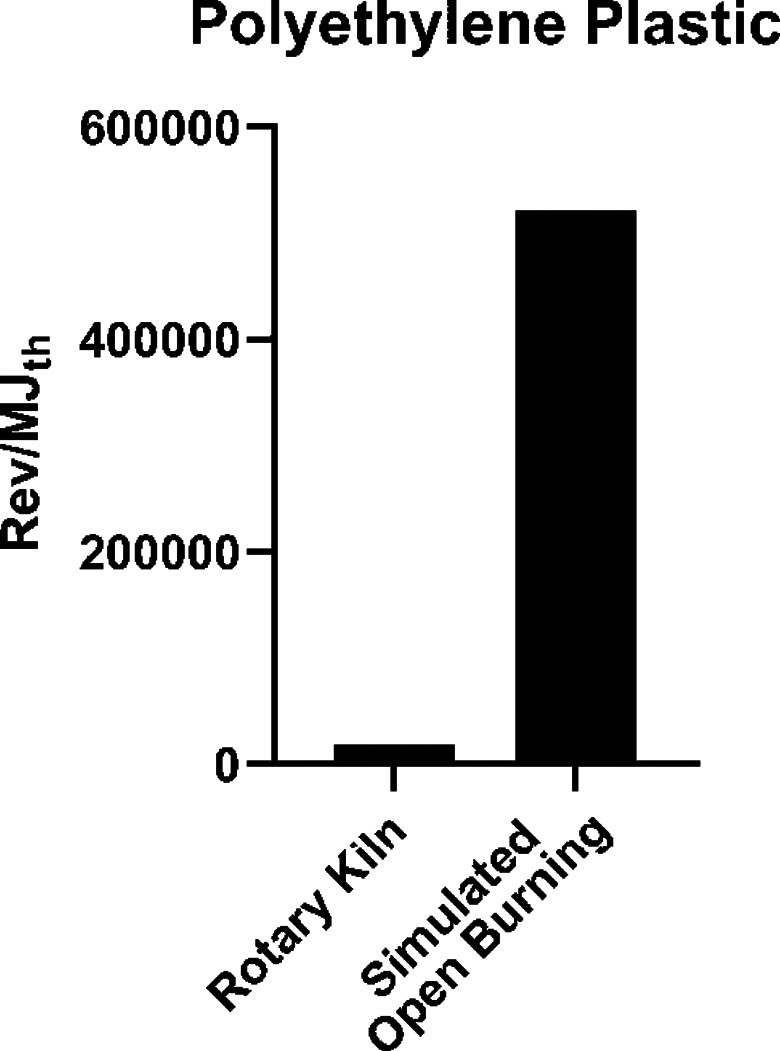
Mutagenicity emission factors in *Salmonella* TA98 + S9 for polyethylene plastic burned either in a rotary kiln (DeMarini et al., 1992) or by simulated open burning (Linak et al., 1989). Error measurements were not provided in these two papers, preventing an analysis of significant differences between the samples

A similar observation is shown in Figure 8 for polyethylene burned two different ways. The rotary kiln experiments involved extremely high temperatures and a short period of time when oxygen levels fell to zero. As the transient polyethylene charge was introduced, there was a short period of time when excess oxygen from the continuous natural gas flame was sufficient to completely oxidize the vaporizing gases. This was followed by an oxygen‐deficient period while evolving gases completely overwhelmed the available oxygen, and finally, as the rate of vaporization slowed, the systems became fuel‐lean, and oxidation returned to near completion. During the period when oxygen dropped to zero, conditions are best described as high temperature pyrolysis, similar to processes used to produce carbon black. PM emitted during the first and third phases of this process were low because the available oxygen and temperatures were sufficient to fully oxidize the evolving gases, and although PM emissions during the second high temperature pyrolysis phase were high, they had low %EOM (see Table 1) and, thus, correspondingly low mutagenicity per MJ_th_. In contrast, the open burning of agricultural plastic reflected the buoyancy and natural convective forces that controlled fuel/air mixing. Excess oxygen was always available (but not necessarily where and when it was needed), temperatures were relatively low, and mixing was poor. Available oxygen was never zero, but incomplete combustion produced massive quantities of PM with high %EOM. This closely describes the other open burning scenarios.

Thus, the fundamental features of good combustion (minimizing kinetic and mass‐transfer limitations by promoting high residence times, temperatures, and turbulence), which are found in well‐designed combustion systems such as utility and industrial boilers, incinerators, and other large engineered systems produce the lowest mutagenicity emission factors, whereas poor combustion conditions (low temperatures and poor mixing of fuel and atmospheric oxygen), which are typical of open burning, produce the highest mutagenicity emission factors. These fundamental principles of combustion can inform environmental and health assessments of combustion emissions; how materials are combusted is perhaps more critical than the material itself in terms of the resulting mutagenicity emission factor.

Another way to consider this issue is to look at an example where different types of fuels were combusted by the identical technology yet produced rather similar mutagenicity emission factors. An example of this is the combustion of 100% petroleum diesel, 100% canola biodiesel, 100% waste vegetable oil biodiesel, or 100% soy biodiesel in an identical 4.3‐kW diesel generator (Table 1). As described by Mutlu et al. (2015c), biodiesel fuels are composed of a relatively small set of fatty acid methyl esters (FAME) that are unique to each bio‐oil. Soy‐based biodiesel, for example, is composed primarily of six, C_17_ and C_19_ straight chain FAME species with 0, 1, 2, and 3 C═C double bonds. Petroleum diesel fuels, in contrast, are defined by a distillate fraction, and typically contain C_10_ to C_22_ compounds with boiling points between 130 and 400°C. Petroleum diesel fuel is a complex mixture of thousands of compounds, including normal and branched parafins, olefins, naphthenes, aromatics, and PAHs. Aromatic compounds typically comprise 20%–50% of petroleum diesel fuels. Despite the considerably different chemical compositions of these fuels, the rev/MJ_th_ or rev/kg fuel each spanned a factor of 8× (<1 order of magnitude). All of the 100% biodiesel fuels had mutagenicity emission factors that were less than half of that of 100% petroleum diesel (Table 1; DeMarini et al., 2019).

## 5 CORRELATIONS BETWEEN MUTAGENICITY AND POLLUTANT EMISSION FACTORS: IMPORTANT ROLE OF PAHs

There are strong correlations (*r* ≥ .9) between the mutagenicity emission factors and pollutant emission factors for a variety of cookstoves burning wood (Champion et al., 2020; Mutlu et al., 2016). These pollutants include total PAHs, PM, PM_2.5_, organic carbon (OC), total hydrocarbons (THC), and methane (CH_4_). Smaller correlations (*r* = ~0.4–0.7) were found between mutagenicity emission factors and pollutant emission factors for oxides of nitrogen (NO_x_), elemental carbon (EC), and CO. Levels of PM‐associated mutagenicity per cubic meter of outdoor air are positively associated with concentrations per cubic meter of NOx, lead, PAHs, CO, nitroarenes (nitro‐PAHs), and sulfur dioxide (SO_2_) (IARC, 2016).

**FIGURE 9:**
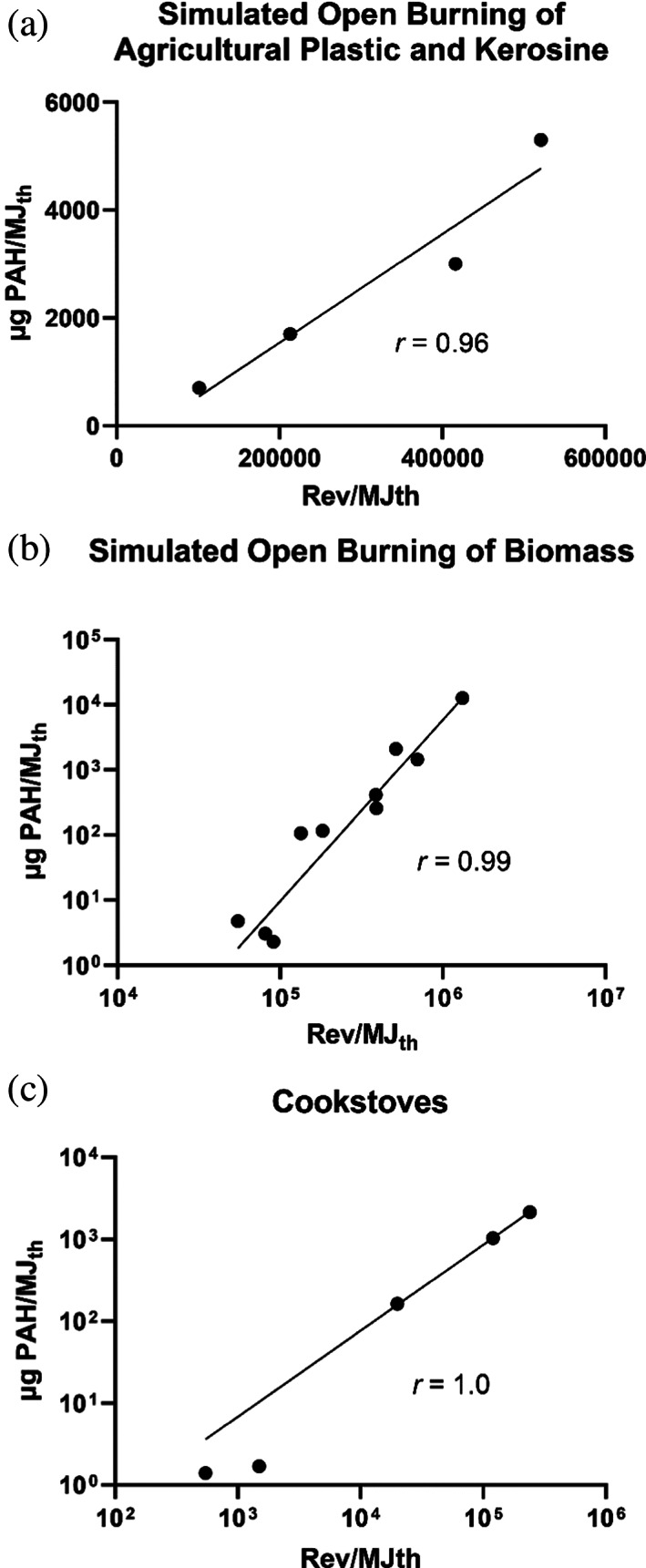
Correlation between mutagenicity and PAH emission factors for (a) agricultural (polyethylene) plastic and kerosene burned in a rotary kiln (Linak et al., 1989), (b) simulated biomass burning (Kim et al., 2018), and (c) cookstoves (Champion et al., 2020; Mutlu et al., 2016); data from Tables 1 and 2

Among these pollutant emission factors, the PAH emission factors were generally the most highly correlated with the mutagenicity emission factors. Table 2 shows the PAH emission factors available for the combustion emissions reviewed here. Comparing these data with the mutagenicity emission factors from Table 1, we found high correlations between mutagenicity emission factors and PAH emission factors for the simulated open burning of agricultural plastic (polyethylene) and kerosene (Figure 9a), simulated open burning of various types of biomass (Figure 9b), and various types of cookstoves burning wood (Figure 9c). However, we did not find a significant correlation between these emission factors for the emissions from the 4.5‐kW diesel generator (Tables 1 and 2).

**TABLE 2:** PAH emission factors for various combustion emissions^a^

Source	μg PAH/g EOM	μg PAH/g PM	μg PAH/m^3^	μg PAH/MJ_th_	mg PAH PM/kg fuel^b^	mg PAH total/kg fuel^c^	References
4.3‐kW Diesel generator							
100% Diesel fuel	8,420	2,190		92			Mutlu et al. (2015a), Mutlu et al. (2015b)
Diesel +20% soy	6,040	1,270		47			Mutlu et al. (2015a), Mutlu et al. (2015b)
Diesel +50% soy	5,920	1,880		72			Mutlu et al. (2015a), Mutlu et al. (2015b)
100% Soy biodiesel	3,160	1,890		57			Mutlu et al. (2015a), Mutlu et al. (2015b)
Cookstoves							
Three‐stone fire		20,070		2,150	31,000		Mutlu et al. (2016)
Natural‐draft stove		16,170		1,030	14,900		Mutlu et al. (2016)
Forced‐draft stove		5,840		160	2,650		Mutlu et al. (2016)
Pellet‐fueled gasifier							
Hardwood		77		1.4	0.026		Champion et al. (2020)
Peanut hulls		257		1.7	0.023		Champion et al. (2020)
Open burning							
Crude oil		5,110		7,190	290	980	DeMarini et al. (2017), Gullett et al. (2017)
Tires			3,400,000	9,370,000,000			DeMarini et al. (1994a), Lemieux et al. (1998)
Simulated open burning							
Kerosene			5	3,000			
Agricultural plastic^d^							
Clean pile			71	5,300			Linak et al. (1989)
Clean forced air			22	1,700			Linak et al. (1989)
Used pile			8	700			Linak et al. (1989)
Used forced air			5	400			Linak et al. (1989)
Red oak							
Smoldering		85		257			Kim et al. (2018)
Flaming		220		5			Kim et al. (2018)
Peat							
Smoldering		640		1,450			Kim et al. (2018)
Flaming		140		2			Kim et al. (2018)
Pine needles							
Smoldering		2,490		12,700			Kim et al. (2018)
Flaming		105		3			Kim et al. (2018)
Pine							
Smoldering		492		2,110			Kim et al. (2018)
Flaming		5,280		116			Kim et al. (2018)
Eucalyptus							
Smoldering		95		412			Kim et al. (2018)
Flaming		5,140		107			Kim et al. (2018)

^a^ Values are from the cited papers, or we calculated the values from data in the papers; values are expressed as 2–3 significant figures.

^b^ PAH concentration derived from organic extract of PM.

^c^ PAH concentration derived from organic extracts of PM + organic extracts of XAD.

^d^ Data are the sum from the prefilter and postfilter.

This is consistent with the discussion above in that these different diesel fuels were combusted in the same diesel engine. PAH and mutagenicity values for the three biodiesel blends each vary by factors of two, and those for petroleum diesel are another factor of two larger. There is a slight trend of increasing mutagenicity with increasing PAH, but with a *p* value of .33, the correlation is not significant. This behavior may be related to minor fuel effects, but our hypothesis that combustion technologies rather than fuel compositions are most important is supported by this lack of correlation. These analyses add to those discussed below involving the strain/S9 specificity of *Salmonella*, mutation spectra, and bioassay‐directed fractionation and chemical analyses, which show that PAHs play an important role in the mutagenicity and carcinogenicity of combustion emissions.

## 6 MUTATION SPECTRA OF ORGANICS FROM AIR AND COMBUSTION EMISSIONS

The important role of PAHs, aromatic amines, nitro‐PAHs, and so forth, in the mutagenicity and carcinogenicity of air and combustion emissions is reflected in the mutation spectra, that is, the types of mutations, induced by such complex mixtures in the base‐substitution strain TA100 of *Salmonella*. Thus, G to T is the predominant base substitution induced in the presence of S9 by PM from air and its base/neutral fraction (DeMarini et al., 1994b) as well as by cigarette smoke condensate (DeMarini et al., 1995) and smoky coal (Granville et al., 2003), reflective of PAHs and aromatic amines (DeMarini, 2000; IARC, 2010b; Kucab et al., 2019). G to T base substitutions are the primary base substitutions induced by PAHs and aromatic amines (DeMarini, 2000). In contrast, the primary classes of inferred base substitutions induced in the absence of S9 by municipal waste incinerator emissions and its fractions are G to A and G to C, along with G to T (DeMarini et al., 1996). Incinerator emissions exhibit direct‐acting mutagenicity (i.e., are mutagenic in the absence of S9) because they contain nitro‐PAHs, which are direct‐acting mutagens. Thus, these emissions were tested for mutagenicity and their mutation spectra in the absence of S9. The G to A, G to C, and G to T mutations noted above reflect the types of mutations induced by various types of nitro‐PAHs, which are direct‐acting mutagens in these emissions (DeMarini et al., 1996). Nitro‐PAHs induce more of these classes of mutation than does the PAH B[*a*]P or the aromatic amine 4‐aminobiphenyl (DeMarini, 2000; DeMarini et al., 1996; Kucab et al., 2019; Levine et al., 1994).

Mutation spectra in tumors associated with exposure of people to these combustion emissions are consistent with the mutation spectra of organic extracts of these emissions in experimental systems. For example, G to T base substitution is the primary class of mutation found in the *TP53* and *KRAS* genes in lung tumors of women whose lung cancer is associated with exposure to smoky coal PAHs (DeMarini et al., 2001), and it is the primary mutation induced in *Salmonella* by a condensate of smoky coal emissions (Granville et al., 2003). Likewise, nitro‐PAH mutations are also found in the lung tumors of non‐smokers (COSMIC, 2021; Zhang et al., 2021), whose lung cancer may be associated with exogenous exposures such as diesel exhaust where nitro‐PAHs are a primary cause of mutagenicity (DeMarini et al., 2004; Mutlu et al., 2013). This is consistent with the general observation that a mutagen induces the same primary class of base substitution across species, from bacteria to humans (DeMarini, 2000).

## 7 ROLE OF THE GAS PHASE ON THE MUTAGENICITY OF COMBUSTION EMISSIONS AND AIR POLLUTION

There are ~12 studies on the mutagenicity of the gas phase of ambient air, most of which evaluated organic extracts of XAD or polyurethane foam (PUF) in the *Salmonella* mutagenicity assay (IARC, 2016). In general, these extracts exhibited both direct and indirect (S9‐dependent) mutagenic activity, whereas organic extracts of most PM samples are generally more mutagenic in the presence of S9 rather than in the absence of S9. The contribution of the gas phase to the mutagenicity of air is less than or equal to that contributed by PM. No studies have evaluated the mutagenicity of semi‐volatiles (as measured from organic extracts of XAD or PUF) from combustion emissions; however, Linak et al. (1989) evaluated some chemical characteristics of XAD extracts from the simulated open burning of agricultural plastic (polyethylene sheets) or kerosene, used to initiate combustion, and evaluated separately as a blank. As reviewed by Riedel et al. (2018) and Zavala et al. (2018), an additional 12 studies have evaluated the mutagenicity and mutation spectra of simulated gas phase atmospheres generated by the interaction of volatile organic compounds (VOCs) with simulated sunlight (UV) in smog chambers. Although the gas phase of air is mutagenic, all risk assessments for lung cancer from polluted air (Hamra et al., 2014; IARC, 2016) or combustion emissions such as diesel exhaust (IARC, 2014) are based solely on the PM fraction.

## 8 CONCLUSIONS

Extensive studies have shown that combustion emissions are universally mutagenic and carcinogenic. The carcinogenic potencies on mouse skin of the EOM from the PM of combustion emissions are highly associated with the unit lung cancer risk in humans exposed to those emissions. These carcinogenic potencies span 3 orders of magnitude both in mouse and humans. Consistent with this is the mechanistic evidence from hundreds of studies showing that people exposed to ambient air pollution, especially air impacted with traffic exhaust, have elevated levels of a variety of genotoxicity biomarkers and an increased risk for lung cancer.

Despite the diverse chemical compositions of EOM and PM from a wide array of air samples worldwide and from diverse combustion emissions, the mutagenic potencies of EOM and PM span only 1–2 orders of magnitude, whereas the mutagenicity of air expressed per cubic meter and the mutagenicity emission factors (mutagenicity per mass of fuel burned) for a wide array of combustion emissions span >5 orders of magnitude. Studies of 50 combustion emissions involving a variety of fuels and technologies for combusting those fuels show that the mutagenicity emission factor is most impacted by the technologies used to burn the fuels (i.e., how materials are burned), rather than what materials are burned. This is not to say that fuel composition does not influence the mutagenicity of the resulting emissions, but that the effects of fuel composition are considerably less important than the combustion technology used. Consequently, the amount of PM rather than the mutagenic potency of the PM itself, is the most critical factor impacting the mutagenicity of air per cubic meter or the mutagenicity emission factor of combustion emissions.

Thus, highly efficient combustion, as seen in industrial and utility boilers and other large engineered systems that minimize kinetic and mass transfer limitations by promoting high temperatures and turbulent mixing of fuel and oxygen, have the lowest mutagenicity emission factors, whereas open burning conditions, such as wildfires, structural fires, and burn pits, which are characterized by relatively low temperatures and poor mixing of fuel and oxygen, have the highest mutagenicity emission factors. These mutagenicity emission factors are highly correlated with PAH emission factors and are relatively independent of the type of material being combusted.

Although chemically diverse, the universal presence of PAHs, and frequently of nitro‐PAHs and aromatic amines, in PM account for much of the mutagenic and carcinogenic activity of PM‐associated air pollution and combustion emissions. This is reflected in the mutation spectra of these classes of compounds and of the EOM from these emissions in experimental systems and in the mutations found in tumors in humans whose cancers are associated with exposure to these emissions (such as cigarette smoke or smoky coal emissions); the primary base substitution being G to T mutations, reflective of PAHs. Although only a few studies have examined the mutagenicity of the gas phase of air pollution, studies of real‐world samples and of atmospheres generated by the interaction of VOCs with simulated sunlight (UV) in smog chambers show that the gas phase is also mutagenic. Collectively, these data provide some fundamental principles to guide environmental and public health assessments of air pollution and combustion emissions.

## CONFLICTS OF INTEREST

The authors declare no conflicts of interest.

## AUTHOR CONTRIBUTIONS

David M. DeMarini and William P. Linak both conceived and wrote this paper.
